# Complete Genome Sequence of Deformed Wing Virus Isolated from *Vespa crabro* in Italy

**DOI:** 10.1128/genomeA.00961-17

**Published:** 2017-10-05

**Authors:** Mario Forzan, Antonio Felicioli, Simona Sagona, Patrizia Bandecchi, Maurizio Mazzei

**Affiliations:** Department of Veterinary Science, University of Pisa, Pisa, Italy

## Abstract

In this article, we document the first isolation of a replication-competent deformed wing virus from *Vespa crabro* in Italy. Although the virus has never been isolated from this insect, the sequence of this virus shows a strong sequence homology with isolates obtained from *Apis mellifera*, which is considered its natural host.

## GENOME ANNOUNCEMENT

Deformed wing virus (DWV) is a viral pathogen of honeybees that is responsible for asymptomatic infection (covert) that can become clinically manifested (overt) in a stressful situation such as high infestation of the mite *Varroa destructor*, which is the biological vector of DWV. Clinical symptoms in honeybees include early death of pupae and deformed wings, shortened abdomen, and cuticle discoloration in adult bees that result in an inability to fly (1).

The virus is assigned to the *Iflavirus* genus belonging to the family *Picornaviridae*. Its genome consists of a 10-kb positive single-stranded RNA with a single open reading frame (ORF) flanked by a long 5′ untranslated region (5′ UTR) and a short, highly conserved 3′ UTR terminating with a 3′ poly(A) tail. DWV sequences available from various parts of the world share 98% to 99% identity, which is consistent with the suggested recent global spread of the virus and evolutionary divergence that is still limited (2).

Here we report the complete nucleotide sequence of a replication-competent DWV strain isolated from a *Vespa crabro* queen with deformed wings collected in October 2016 in Italy.

The viral RNA was extracted from *V. crabro* abdomen using an RNeasy tissue kit (Qiagen, Hilden, Germany) eluted in 30 µl as previously described (3). Viral cDNA was synthetized by use of a Superscript II reverse transcriptase kit (Thermo Fisher Scientific, Waltham, MA, USA) using oligonucleotide dT primers.

A total of 14 overlapping amplicons covering the entire viral genome were generated using Kapa HF *taq* polymerase (Kapa Biosystems, Boston, MA, USA) and sequenced by BMR Genomics (Padova, Italy). Sequences were then assembled using Bioedit software (4). Sequence analyses were performed by MEGA software and Simplot software.

The complete sequenced genome is 10,135 nucleotides (nt) in length, comprising 2 UTRs. The long UTR at the 5′ end spanning from nt 1 to 1136 contains the putative internal ribosome entry site (IRES). The ORF spans from nt 1137 to 9817 and encodes the viral polyprotein. A 317-nt-long UTR is present at the 3′ end, including a 31-nt poly(A) tail.

The DWV *V. crabro* sequence scored 97.9% nucleotide identity with previously reported DWV isolated in Italy from *Apis mellifera* L. (DWV-it, GenBank accession no. AJ489744) (2). Simplot analysis indicated a 98.22% nucleotide homology to DWV type A virus (GenBank accession no. NC_004830), which was suggested as the less virulent circulating strain (5).

We present the first whole-genome sequence of a DWV type A isolated from a symptomatic *V. crabro* collected in Italy. This finding demonstrates for the first time the presence of DWV in a new species belonging to the family *Vespidae*. Sequence analysis showed a strong homology to DWV circulating viruses isolated from *Apis mellifera*, indicating the adaptation ability of the virus into a new host. This information is important to understand alternative transmission routes of the virus.

### Accession number(s).

The complete genome sequence of deformed wing virus isolate *Vespa*_*crabro*_DWV_PI_2016 has been deposited in the GenBank database under the accession number KY909333.

## References

[1] de Miranda JR, Genersch E 2010 Deformed wing virus. J Invertebr Pathol 103:S48–S61. doi:10.1016/j.jip.2009.06.012. 19909976

[2] Lanzi G, de Miranda JR, Boniotti MB, Cameron CE, Lavazza A, Capucci L, Camazine SM, Rossi C 2006 Molecular and biological characterization of deformed wing virus of honeybees (*Apis mellifera* L.). J Virol 80:4998–5009. doi:10.1128/JVI.80.10.4998-5009.2006. 16641291 PMC1472076

[3] Hall T 1999 BioEdit: a user-friendly biological sequence alignment editor and analysis program for Windows 95/98/NT. Nucleic Acids Symp Ser 41:95–98.

[4] Mazzei M, Carrozza ML, Luisi E, Forzan M, Giusti M, Sagona S, Tolari F, Felicioli A 2014 Infectivity of DWV associated to flower pollen: experimental evidence of a horizontal transmission route. PLoS One 9:e113448. doi:10.1371/journal.pone.0113448. 25419704 PMC4242645

[5] McMahon DP, Natsopoulou ME, Doublet V, Fürst M, Weging S, Brown MJF, Gogol-Döring A, Paxton RJ 2016 Elevated virulence of an emerging viral genotype as a driver of honeybee loss. Proc Biol Sci 283:443–449. doi:10.1098/rspb.2016.0811. PMC4936039 27358367

